# Tapping into the vocal learning and rhythmic synchronization hypothesis

**DOI:** 10.1186/s12868-024-00863-2

**Published:** 2024-11-06

**Authors:** Constantina Theofanopoulou

**Affiliations:** 1 Rockefeller University, New York, NY USA; 2 Center for Ballet and the Arts, New York University, New York, NY USA; 3 Drexel University, Philadelphia, PA USA

**Keywords:** Vocal learning, Rhythmic synchronization, Rhythm entrainment, Beat synchronization, Language, Speech, Dance

## Abstract

In this article, I present three main points that could benefit the “vocal learning and rhythmic synchronization hypothesis”, encompassing neurogenetic mechanisms of gene expression transmission and single motor neuron function, classification of different behavioral motor phenotypes (e.g., spontaneous vs. voluntary), and other evolutionary considerations (i.e., the involvement of reward mechanisms).

The “vocal learning and rhythmic synchronization hypothesis” (VLRSH) [1], formulated by Ani Patel, aims to explain how the evolution of advanced *vocal* learning in humans and parrots may have resulted in their ability to synchronize *nonvocal* movements to a rhythmic beat. I believe this hypothesis provides one of the most promising avenues for linking the evolution of two core components (i.e., vocal learning and rhythmic synchronization) that are necessary for complex sensorimotor behaviors such as speech, song, and dance. Earlier versions of this hypothesis [2, 3] have been influential in shaping my research agenda, where I currently focus on studying the brain pathways involved in speech and dance production and learning in humans. In this version of the VLRSH [1], Patel delves deeper into the neurobiological mechanisms, proposing that the evolution of a strong integration between auditory regions and *vocal* dorsal premotor regions in ancestral humans (via the laryngeal pitch control pathway) involved gene regulation changes which fortuitously enhanced the strength of neural connections between auditory and *nonvocal* dorsal premotor regions near the vocal dorsal premotor regions.

Ani Patel has invested significant effort in synthesizing existing literature to construct a hypothesis that is both robust and testable. While acknowledging the solidity of Patel’s work that has uniquely inspired my research, I identify areas within the hypothesis that merit further refinement and exploration. In this commentary, I intend to address three specific points derived from Patel’s hypothesis. Firstly, I will dig into the neural mechanisms and suggest an alternative or complementary scenario for the gene regulation changes in neighboring brain regions that, according to Patel [1], could have led to the enhancement of the brain pathways needed for rhythmic synchronization. Secondly, I advocate for a more profound understanding of what constitutes “voluntary”, “involuntary”, “reflexive” or “spontaneous” movements, whether *vocal* or *nonvocal*, as a beneficial addition to the hypothesis. Thirdly, I will present my perspective on an additional parameter that Patel suggests could have contributed to the human and parrot ability to dance: their “craving for social interaction and a strong sensitivity to social reward [4]”.

## Adjacent and/or overlapping motor circuits for vocal learning and rhythm synchronization

One of the fundamental tenets of the VLRSH is the notion that gene regulation changes in specific neurons, particularly those engaged in the integration between auditory and *vocal* dorsal premotor regions, triggered alterations in the gene regulation of adjacent or nearby neurons involved in the integration between auditory and *nonvocal* dorsal premotor regions. However, the mechanistic details of how neurons can directly impact the gene expression of neighboring neurons remain unclear. While certain mechanisms exist, such as neurotransmitter signaling influencing the electrical activity of the receiving neuron, the release of neurotrophic factors from one neuron to another, or activity-dependent plasticity, none of these mechanisms explain the proposed “infection” of gene expression to adjacent neurons posited by the VLRSH.

While formulating my working hypothesis for studying in tandem the speech and dance brain pathways in humans, I was able to find only limited instances of research demonstrating the feasibility of gene expression transmission from one neuron to another. For instance, experiments involving Xenopus tectal neurons revealed that overexpressing Candidate Plasticity Gene 15 (CPG15) not only enhanced dendritic outgrowth and synapse maturation in the directly overexpressed neurons but also influenced these characteristics in adjacent neurons, potentially through intercellular interactions [5, 6]. Another set of experiments in transgenic animal models of neurodegenerative diseases also identified the “spread” of gene expression properties in neighboring neurons [7]: in these models, the accumulation of Tau in neurons expressing a transgene resulted in tau aggregates developing in adjacent neurons lacking the transgene but receiving projections from transgene-expressing neurons, possibly through a trans-synaptic prion-like mechanism [7]. Additionally, in neuropathic pain mouse models, experimental injury to a set of neurons in the dorsal root ganglion led to differential gene regulation in the injured neurons also affecting gene regulation in nearby intact neurons, possibly through the activation of intracellular second messenger systems inducing immediate early genes (IEGs) controlling expression changes in other genes [8]. While these studies offer insights into potential mechanisms for studying the VLRSH, the direct transmission of changes in gene expression from one neuron to adjacent neurons remains a concept not firmly established.

Testing this tenet of the VLRSH hypothesis is challenging, even with advanced spatial transcriptomic tools like single-nucleus RNA sequencing that enable us to identify gene expression patterns in single nuclei of individual neurons. One approach to test the idea of direct transmission of gene expression changes from one neuron to adjacent neurons would involve manipulating (e.g., knocking down) the expression of a gene in one or more neurons and conducting single-nucleus RNA-sequencing in both manipulated and adjacent neurons. Confirmation of the VLRSH tenet would require observing, for example, a significant downregulation trend for the knocked-down gene in adjacent neurons of mutant animals, along with significant similarities in the regulation patterns of pathway-related genes, compared to equivalent comparisons of analogous neurons in wild-type animals. Similar approaches are anticipated to unveil the dynamic and intricate mechanisms of neuron-to-neuron interactions.

An alternative or complementary hypothesis I wish to put forward is that it might be not the adjacent, but the *very same neurons* being responsible for both the abilities of vocal learning and rhythmic entrainment. In other words, I hypothesize that some of the same neurons in the primary motor cortex (M1) that are responsible for the precise and rhythmic control of *vocal* muscles might also project to other *nonvocal* movement muscles, such as hand or leg muscles. Is there any evidence supporting the idea of M1 neurons projecting to distinct motor muscles? Recent strong evidence reveals that injecting a pseudorabies virus with different fluorophores into both the cricothyroid (laryngeal) muscle and the extensor carpi radialis (forelimb) muscles stained overlapping single neurons in the mouse M1 [9]. Remarkably, this phenomenon was not confined to a small subset of neurons; instead, the proportions of co-labeled cortical neurons spanned from 5.3 to 25% across M1.This suggests that, even in a species like the mouse with limited vocal flexibility [10], the same neurons controlling laryngeal muscles for vocal communication also project to forelimb muscles for various hand movements. In another study [11], which utilized orthograde axonal transport tracing methods in rats, injections focused on the motor face cortex (jaw, lip, and tongue areas) resulted in the same terminal fields of the basilar pontine nuclei of the brainstem where limb sensorimotor cortices’ injections also terminated. A more recent study [12] in monkeys identified that M1 cells were not specifically tuned to individual muscles but exhibited a more “functional” activity, e.g., specific to various functions of different muscles, like synergist, fixator, or antagonist functions (see also [13]). These collective studies challenge the notion of a one-to-one relationship between a muscle and a motor neuron. If this holds true, it suggests that the same motor neurons that evolved the ability to finely and rhythmically control laryngeal muscles may have also employed this ability in their projections to other muscles involved in different body movements.

Turning our attention to humans, is there any evidence indicating that the same motor neurons project to different muscles? Contrary to the traditional view presented by Penfield’s motor homunculus [14], where each muscle type corresponds to a specific part of M1, recent evidence [15, 16] challenges the notion of a “one muscle, one motor neuron” principle and suggests that motor representations in human M1 are organized based on different principles, such as the “like attracts like” principle, where effectors for movements that frequently occur together or serve similar functions tend to cluster or be spatially organized together. Arguably the most compelling evidence for an overlap of *vocal* and *nonvocal* projecting neurons arises from a series of studies [17, 18] where intracortical electrodes were implanted in the conventionally identified “hand” area of the dorsal M1 and showed significant firing rate changes during speech. Notably, these studies not only demonstrated that the “hand” area encodes specific spoken phonemes, but also achieved high-accuracy decoding of these phonemes using both intracortical multiunit spikes and local field potential power. Recent functional Magnetic Resonance Imaging (fMRI) studies by Gordon et al. [19] showed that human M1 comprises motor-specific regions intermingled with so-called “inter-effector” integrating areas lacking movement specificity. In our ongoing work in preparation, we are leveraging this evidence by comparing fMRI activation maps of various speech-related laryngeal movements with rhythmic hand, leg, hip, and other movements, while excluding relevant activity from control tasks, such as non-speech laryngeal movements (e.g., coughing or laughing) and non-rhythmic body movements. Our data suggest the involvement of both overlapping and adjacent brain regions in speech (vocal) laryngeal movements and other rhythmic (dance) body movements. Further exploration will unfold the neuronal-level processes in humans, shedding light on whether mechanisms within the same and/or adjacent neurons contribute to fine rhythm entrainment abilities in body muscles beyond the larynx. Future investigations into the auditory-to-motor integration pathways proposed by Patel [1] in humans are also expected to provide insights into the connectivity patterns supporting the motor cortex-based scenarios I proposed here.

Given the temporal or spatial limitations of most neuroimaging techniques, such as fMRI and electroencephalography (EEG), in testing my hypothesis regarding the overlap of neurons in the M1 responsible for both *vocal* and *nonvocal* muscle control, I suggest using advanced neural recording technology, neuropixels, to analyze the response of neurons across M1. Neuropixels, with nearly 1,000 electrode sensors in an area about the size of a human eyelash, allow recording individual neuron activity in awake participants. Recently, Leonard et al. [20] recorded from 685 neurons in the superior temporal gyrus, a critical region for speech perception, and found single neurons encoding various speech sound cues, such as features of consonants and vowels, relative vocal pitch, onsets, and amplitude envelope. To test my hypothesis, an experiment would require recording single neuron activity in different M1 layers, specifically the laryngeal and hand motor areas, while participants speak or tap their fingers rhythmically to an internal or external beat. Confirming the hypothesis would indicate overlap in neurons activated during speech and finger tapping cues, such as pitch and tempo, respectively, while refuting it would suggest different neurons for each activity. Intracortical implant arrays detecting single-unit activity could also be applied to similar protocols for further investigation.

## Towards a framework for classifying different types of movements

Shifting focus, I propose that the VLRSH could benefit from a clearer framework outlining the specific types of *vocal* and *nonvocal* movements suitable for studying the hypothesis’s confirmation or falsification. This could be achieved with a precise definition and categorization of movements referred to, in Patel’s manuscript, as “spontaneous”, “involuntary”, “voluntary”, or “reflexive”. For example, Patel refers to a study [21] on rats passively exposed to music, where small head movements around the times of beats are interpreted by him as “involuntary” or “reflexive”. In motor neuroscience, the term “spontaneous” is often used interchangeably with these terms. However, Patel specifies in the VLRSH manuscript [1] that “spontaneous” refers instead to rhythmic movements occurring “without reliance on formal training”. I interpret the latter as referring mostly to whether these rhythmic movements are “innate” or “learned”, rather than “spontaneous” (sensu “reflexive”), “reflexive”, “involuntary” or “voluntary”. This interpretation gains support from Patel’s subsequent passages [1], where he discusses a possible developmental period in which children acquire the ability to synchronize predictively and sporadically to a tempo. This suggests to me that, for Patel, “spontaneous”, in the context of rhythmic synchronization, primarily denotes a form of natural (not formal) learning, akin to first language acquisition, a perspective with which I agree. While recent evidence aligns with this perspective (e.g., [22]), the term “spontaneous” might not accurately convey the intended message, particularly given its usage as synonymous with “involuntary” or “reflexive” in the broader neuroscience field.

Interestingly, we recently shared a preprint [23] introducing a relevant framework that delves into a more refined classification of human vocalizations (i.e., laryngeal muscle movement) that is built upon the terms “innate”, “learned”, “spontaneous” and “volitional”. Traditionally, scientists have categorized human vocalizations into “innate” and “learned”, with innate encompassing vocalizations like coughing, laughter, sneezing, and yawning, and learned including, basically, speech. The general understanding has been that innate vocalizations are predominantly produced “spontaneously”, without any volitional vocal control [24]. However, a caveat arises from findings indicating that these so-called “innate” vocalizations often require intentional modulation of vocal output [25], which is evident in the differences of these vocalizations across different cultures and languages (e.g., laughter), with these differences being readily discernible by the listeners [26]. Adding complexity, so-called “innate” vocalizations can be either “spontaneous” (e.g., laughter evoked by tickling) or “volitional” (e.g., fake laughter). Relevant accounts [27] attempting to categorize honest vs. fake “Duchenne” smiles (not laughter) further highlight the learnability component of fake smiles, making one wonder if they should be categorized as innate in the first place. Similarly, for laughter, there could be types of laughter that are full products of learning and not volitional imitations of one’s innate laughter. Moreover, vocalizations typically considered “learned” may be emitted in contexts that are more “spontaneous” than “volitional” (e.g., exclaiming “Ouch!” in response to pain).

Based on these insights, we proposed a continuum for human vocalizations, ranging from innate to learned on one axis and from spontaneous to volitional on the other [23]. Within this continuum, one can find innate vocalizations produced spontaneously, such as exclaiming “Ah!” in response to pain, or innate vocalizations produced volitionally, as seen in “ironic” or “fake” laughter (“Ha, ha!”). Similarly, there are learned vocalizations in both (volitional and spontaneous) categories, with examples like volitional expressions such as “Thanks!” in a conversation, or learned vocalizations produced spontaneously, like exclaiming “Ouch!” in response to pain. While extending this framework to classify other body movements (e.g., hand or leg) would require a separate discussion, I am optimistic that this continuum can serve as a valuable foundation for a deeper understanding of where rhythmic movements may fall within this spectrum.

## Vocal learning, rhythm synchronization and social reward

According to Patel [1], another contributing factor to the ability for rhythmic movement observed in humans and parrots could be attributed to their “craving for social interaction and a strong sensitivity to social reward [4]”. I wholeheartedly agree with Patel and believe that this factor may play an even more pivotal role in the ability of humans and parrots to synchronize their body movements to a beat. Here, I would like to explore further the framework through which we examine concepts like “social interaction” and “social reward”. Typically, these terms are often interpreted within a Pavlovian framework, where the reward is perceived as *external*. For instance, as Patel [1] notes, “Snowball and other pet parrots that move rhythmically to music often get attention and praise from their owners for this behavior”. However, Schmidt and Kaplan [28]raise a significant question: “Does Snowball dance by himself, even when no one is watching?”. In other words, does Snowball engage in dance without any *external* reward? Is the act of dancing *internally* rewarding for Snowball?

The field of neuroscience has yet to establish a clear distinction between *external* and *internal* rewards, along with the corresponding brain pathways underlying them. It is still unknown whether this lack of clarity in their distinction is due to an absence of evidence or a genuine absence of differences between these two types of reward pathways. Examining evidence from songbirds, it is noteworthy that dopamine is implicated in both what we typically label as *external* and *internal* rewards. For instance, in male zebra finches, dopamine levels in Area X of the striatum are higher when they sing to attract females (*external* reward) compared to when they sing in isolation (*internal* reward) [29]. This increase may be attributed to differential activity of the reuptake transporter (a noradrenaline transporter in birds) in the Ventral Tegmental Area (VTA) axons projecting to Area X. Blocking this transporter pharmacologically equalizes dopamine levels during undirected singing to those during directed singing [29]. However, the question remains whether these distinctions are sufficient to support distinct *external* (social, sexual) and *internal* reward brain pathways. The picture is not entirely clear, especially considering that VTA dopamine neurons projecting to Area X were also found to encode error-and-reward during undirected singing (i.e., *internal* reward) [30]. Altogether, these studies underscore the necessity for a more nuanced understanding of reward brain pathways as integral components of relevant pathways responsible for auditory perception and motor (vocal or nonvocal) production, as observed in the domains of speech/song and dance.

In a series of studies [31–35], I have presented evidence and proposed a role for oxytocin and dopamine within the vocal learning circuitry. Could these neurotransmitters also contribute to our understanding of the relevant reward mechanisms (*external* and/or *internal*) involved in the rhythmic synchronization abilities of humans and parrots? Studies involving administration of intranasal oxytocin (OT) suggest that OT, compared to placebo, increases synchronized interpersonal movement during dance [36] and during an interactive finger-tapping paradigm [37]. While not directly related to dance but pertinent to a crucial component of dancing, music perception has been linked to increases in both oxytocin [38, 39] and dopamine [40]. It would be intriguing to test whether beat synchronization would increase in parrots after OT administration. Further research is essential to unravel the entire complexity of the reward neurocircuitry and explore potential differences in *external* versus *internal* reward mechanisms during dancing, especially considering instances when individuals dance alone, without an audience.

In conclusion, I believe that all the above remarks can only highlight how promising the VLRSH is. Moving forward, conducting behavioral studies that simultaneously explore different species’ abilities for vocal learning and rhythm entrainment is crucial to either validate or challenge the VLRSH. Complementary neural tracing, single-unit recordings, and neuroimaging studies are essential to elucidate the underlying brain pathways. Spatial transcriptomic analyses can provide more targeted insights into the regulation profiles of neurons involved in vocal learning, rhythmic movement, or both. Beyond the confines of this hypothesis, gaining a deeper understanding of how gene expression may propagate from one neuron to another is imperative for the field. Additionally, there is much more to discover regarding the organization of regions like the primary motor cortex and whether motor neurons exhibit a more muscle-specific, “function”-specific, or other organization. Lastly, developing clearer frameworks and working hypotheses to define terms used for specific behaviors, such as distinguishing between spontaneous and volitional movements or internal and external rewards, is a necessary step forward for the field.

## Data Availability

No datasets were generated or analysed during the current study.
